# Cytokine gene polymorphisms are not associated with anti-PvDBP, anti-PvAMA-1 or anti-PvMSP-1_19_ IgG antibody levels in a malaria-endemic area of the Brazilian Amazon

**DOI:** 10.1186/s12936-016-1414-3

**Published:** 2016-07-19

**Authors:** Adriana A. C. Furini, Marcela P. Capobianco, Luciane M. Storti-Melo, Maristela G. Cunha, Gustavo C. Cassiano, Ricardo Luiz D. Machado

**Affiliations:** Department of Dermatology, Infectious and Parasitic Diseases, São José do Rio Preto Medical School, São José do Rio Preto, São Paulo, Brazil; Department of Biology, São Paulo State University, São José do Rio Preto, São Paulo, Brazil; Laboratory of Molecular Genetics and Biotechnology, Department of Biology, Federal University of Sergipe, São Cristóvão, SE Brazil; Laboratory of Microbiology and Immunology, Institute of Biological Sciences, Federal University of Pará (Universidade Federal do Pará-UFPA), Belém, State of Pará (PA) Brazil; Laboratory of Tropical Diseases-Department of Genetics, Evolution and Bioagents, Universidade de Campinas, Campinas, SP Brazil; Laboratory of Basic Research in Malaria, Section of Parasitology, Evandro Chagas Institute, Belém, PA Brazil

**Keywords:** IgG antibody, *Plasmodium vivax*, TNF, IFNG, IL10

## Abstract

**Background:**

The immune response against *Plasmodium vivax* immunogenic epitopes is regulated by pro- and anti-inflammatory cytokines that determine antibody levels and class switching. Cytokine gene polymorphisms may be responsible for changes in the humoral immune response against malaria. The aim of this study was to evaluate whether polymorphisms in the *TNFA*, *IFNG* and *IL10* genes would alter the levels of anti-PvAMA1, PvDBP and -PvMSP-1_19_ IgG antibodies in patients with vivax malaria.

**Methods:**

Samples from 90 vivax malaria-infected and 51 uninfected subjects from an endemic area of the Brazilian Amazon were genotyped using polymerase chain reaction-restriction fragment length polymorphism (PCR–RFLP) to identify polymorphisms of the genes *TNFA* (−1031T > C, −308G > A, −238G > A), *IFNG* (+874T > A) and *IL10* (−819C > T, −592C > A). The levels of total IgG against PvAMA1, PvDBP and PvMSP-1_19_ were determined using an enzyme-linked immunosorbent assay (ELISA). Associations between the polymorphisms and the antibody response were assessed by means of logistic regression models.

**Results:**

No significant differences were found in the levels of IgG antibodies against the PvAMA-1, PvDBP or PvMSP-1_19_ proteins in relation to the studied polymorphisms.

**Conclusions:**

Although no associations were found among the evaluated genotypes and alleles and anti-merozoite IgG class *P. vivax* antibody levels, this study helps elucidate the immunogenic profile involved in the humoral immune response in malaria.

**Electronic supplementary material:**

The online version of this article (doi:10.1186/s12936-016-1414-3) contains supplementary material, which is available to authorized users.

## Background

Early diagnosis, prompt and effective treatment, the use of mosquito nets impregnated with long-acting insecticides and residual intradomiciliary spraying are the main malaria control measures [1] and have resulted in a reduction in the transmission and number of cases of malaria in Brazil. However, this disease remains one of the most prevalent infections in tropical countries, with 214 million clinical cases/year and approximately 438,000 deaths [2]. In Brazil, *Plasmodium vivax* is the aetiologic agent in 85 % of cases, and 99.9 % of cases occur in the Brazilian Amazon region [2].

The different clinical manifestation patterns of malaria may be related to host and agent genetic factors, age and ethnicity as well as the involvement of these factors in the immune response and parasitaemia and antibody levels [3–5]. IgG antibodies play a protective role against parasite invasion in the erythrocytic cycle of *Plasmodium* [3, 6]. For *Plasmodium knowlesi,* anti-PvAMA-1 monoclonal antibodies have been shown to be capable of inhibiting merozoite invasion in vitro [7]. PvDBP antibodies inhibit interactions with the Duffy antigen receptor for chemokines (DARC) in erythrocytes [8], and antibodies produced against PvMSP-1_19_ have been shown to prevent merozoite invasion in vitro [9]. The synthesis of immunoglobulins is complex and depends on the process of antigen presentation by B lymphocytes (BL) to TCD4 lymphocytes and the involvement of co-stimulatory molecules and their receptors [10].

The production, levels and switching of antibody classes is regulated by pro- and anti-inflammatory cytokines [3, 6, 11, 12]. IFN-γ can negatively modulate the humoral immune response, thus interfering with circulating antibody levels [13] and increasing IgG2 production. TNF appears to be important in the development of the humoral response as an autocrine growth factor for B cells [6]. Among the anti-inflammatory cytokines, IL-10 may participate in negative immunomodulation of the Th1-type response [14, 15] in addition to inducing immunoglobulin synthesis [6].

Many genetic variants are responsible for minor changes in the immune response in malaria [11, 12]. Previous studies in Brazil with vivax malaria have evaluated factors associated with genetic variability in the humoral immune response. HLA-DR16 is associated with the IgG antibody response to the *P. vivax* VK247 variant circumsporozoite protein [16]. High levels of MSP-1 antibodies are also associated with HLA-DR3 [17]. In Goianésia do Pará, a municipality located in the Brazilian Amazon, two studies evaluated the effects of polymorphisms in genes involved in the humoral immune response. Cassiano et al. [10] found that the frequency of specific IgG responders against PvAMA-1, PvDBP and PvMSP-1_19_ was associated with polymorphisms in the *BLYS* (−871C > T), *CD40* (−1C > T) and *CD86* (+1057G > A) genes. In contrast, genotypes and haplotypes of the *IL4* gene were not associated with the production of PvAMA-1 antibodies [18]. The aim of this work was to continue the search for the genetic basis of these traits and to evaluate whether polymorphisms in the *TNFA, IFNG* and *IL10* genes alter the levels of anti-PvAMA1, -PvDBP and -PvMSP-1_19_ IgG antibodies.

## Methods

### Study area and subjects

Ninety samples from vivax malaria-infected subjects and 51 samples from uninfected subjects were collected in Goianésia do Pará (03°50′33″S; 49°05′49″ W). The subjects were in a sub-group of individuals previously analysed by Cassiano et al. [10]. The study has evaluated the effect of genetic ancestry on the distribution of polymorphisms in the *TNFA*, *IFNG* and *IL10* genes (unpublished data). No differences were observed in the mean proportion of any ancestry among the different genotypes and haplotypes analysed.

In 2011 and 2012, the numbers of malaria cases were 2856 and 1136, respectively, with 79 % of cases caused by *P. vivax*. Samples were collected at the municipal health center between February 2011 and August 2012. Data were collected by passive detection in Basic Health Units after thick blood film phenotypic diagnosis, but prior to treatment. All patients if malaria were given standard treatment of 1500 mg of chloroquine in 3 days (600, 450 and 450 mg) plus 30 mg of primaquine on the day the diagnosis and on the following 6 days. Individuals infected with *P. vivax* presented for medical care because of clinical symptoms of malaria and they were recruited after diagnosis. The uninfected individuals who sought medical care offered during the study were invited to participate in the study. These individuals experienced the same conditions of exposure to the aetiological agent. All participants or guardians signed the consent form, and the project was approved by the Goianésia do Pará health authorities and by the Research Ethics Committee (CAAE 01774812.2.0000.5415) of the College of Medicine of São José do Rio Preto (Faculdade de Medicina de São José do Rio Preto).

Malaria was diagnosed using thick blood smears stained with Giemsa and subsequently confirmed by nested-PCR with modifications [19]. No uninfected individual was positive in nested-PCR, while five individuals positive for *P. vivax* (thick blood smear) had mixed infection with *Plasmodium falciparum* (by nested-PCR) and were excluded from the study. Parasitaemia was defined as the number of parasites per microlitre of blood after examination of 100 microscopic fields.

Peripheral blood was stored at −20 °C. The examination of polymorphisms in the genes *TNFA, IFNG* and *IL10* was performed via PCR amplification followed by restriction fragment length polymorphism (RFLP) analysis or the amplification of specific alleles (Table 1).

**Table 1:** Polymorphisms, methods, restriction enzymes, primers, and band patterns used for investigation of SNPs in genes *TNFA, IFNG, IL10*

Polymorphisms in gene	SNP	Method enzimas annealing	Primers	Genotype	Reference
*IFN γ* −*183* *G* > *T*	*rs2069709*	*RFLP* (53º) *Eco47I*	FW: 5′-AAT GAT CAA TGT GCT TTG TG-3′ R: 5′-TAA GAT GAG ATG GTG ACAG-3′	TT: 271 pb GT: 271 pb, 164 pb, 107pb GG: 164pb, 107 pb	Suxia Qi et al. [20]
*IFN γ* +*874* *A* > *T*	*rs2430561*	ASO-PCR (53º)	CP: -5′-TCA ACA AAG CTG ATA CTC CA-3′ T : 5′-TTC TTA CAA CAC AAA ATC AAA TCT-3′ A: 5′-TTC TTA CAA CAC AAA ATC AAA TCA-3′	AA: 262 pb (reação A) TT: 262 pb (reação T) AT: 262 pb (reação A, T)	Medina et al. [21]
*TNF α* −*238* *G* > *A*	*rs 361525*	RFLP (60º) *MspI*	FW: 5′-ATC TGG AGG AAG CGG TAG TG-3′ R: 5-AGA AGA CCC CCC TCG GAA CC’ 3′	AA: 156 pb GG: 133 pb GA: 153 pb, 133 pb	Hedayati et al. [22]
*TNFα* −*308* *G* > *A*	*rs 1800629*	RFLP (59º) *NcoI*	FW: 5′-GAG GCA ATA GGT TTT GAG GGC CAT-3′ R: 5′-GGG ACA CAC AAG CAT CAAG 3′	AA: 147 pb GG: 126 pb, 121 pb GA: 147 pb, 126 pb, 21 pb	Asghar et al. [23]
*TNFα* −*1031* *T* > *C*	*rs1799964*	RFLP (57º) *BbsI*	FW: 5′-TAT GTG ATG GAC TCA CCA GGT-3′ R: 5′-CCT CTA CAT GGC CCT GTC TT 3′	TT: 251 pb, 13 pb TC: 251 pb, 180 pb, 71 pb e 13 pb CC: 180 pb, 71 pb e 13 pb	Asghar et al. [23]
*IL*-*10* −*592* *C* > *A*	*rs1800872*	RFLP (54º) *RsaI*	FW: 5′-GGG TGA GGA AAC CAA ATT CTC-3′ R:5′-GAG GGG GTG GGC TAA ATA TC 3′	AA: 240 pb, 77 pb, 36 pb e 08 pb CC: 317 pb,36 pb e 08 pb CA: 317 pb, 240 pb, 77 pb, 36 pb e 08 pb	
*IL*-*10* −*819* *C* > *T*	*rs1800871*	RFLP (54º) *RseI*	FW: 5′-GGG TGA GGA AAC CAA ATT CTC-3′ R: 5′-GAG GGG GTG GGC TAA ATA TC 3′	TT: 270 pb e 91 pb CC: 217 pb, 91 pb e 53 pb TC: 270 pb, 217 pb, 91 pb e 53 pb	

The amplified products were subjected to electrophoresis (100 V/50 min) in 1.5 to 2.5 % agarose gels stained with Gel Red (Biotium, CA, USA) or in ethidium bromide-stained 12.5 % polyacrylamide gels (10 mg/ml) and were visualized using a transilluminator (Biotecnologia-Locus).

### Assessment of the serological response against *Plasmodium vivax*

The levels of IgG class anti-MSP-1_19_, anti-PvAMA-1 and anti-PvDBP antibodies in a larger cohort were previously reported by Cassiano et al. [10]. The analyses were performed using ELISA following previously described protocols [24–26]. A recombinant protein (His6-MSP-1_19_) comprising amino acids 1616–1704 of MSP-1_19_ [24] and domain II of the DBP protein [25] of *P. vivax* (SAll strain) was expressed in *Escherichia coli*. A protein ectodomain (amino acids 43–487) expressed in *Pichia pastoris* was used for PvAMA-1 [26]. The reactivity index (RI) was calculated to define samples as reactive or non-reactive and was determined by dividing the sample OD value by the cut-off point. Samples with RI ≥ 1 were considered positive, and those with RI < 1 were considered negative. The cut-off point was established as the mean OD (plus three standard deviations) of the 40 plasma samples collected from subjects with no history of malaria who were living in São José do Rio Preto, which is located in the interior of the state of São Paulo (a non-malaria-endemic area). The control samples were only used for the determination of serological response.

### Statistical analysis

Statistical analysis was performed using R software v 2.11.1. The genotypic and allelic frequencies of each variant were calculated using the genetics package [27]. This package was used to evaluate Hardy–Weinberg equilibrium deviations using the Chi square test and the linkage disequilibrium between locus pairs was calculated using the D′ parameter. Haplotypes frequencies were estimated using the maximum likelihood method with the EM (expectation–maximization) algorithm, which is part of the haplo stats package [28]. Differences between the proportions of responders and non-responders were evaluated using the Chi square test. A non-parametric Kruskal–Wallis test was used to identify differences in antibody levels and genotypes. *P* values less than 0.05 were considered significant.

## Results

The genotypic frequencies of the six examined single nucleotide polymorphisms (SNPs) are summarized in Table 2. The allelic frequencies of the evaluated SNPs were in Hardy–Weinberg equilibrium. For the *IFNG* gene, allele A was the most frequent, with 67.3 % at position +874T > A. For positions −592C > A and −819C > T of the *IL10* gene, the C allele was most common, with frequencies of 68.8 and 65.6 %, respectively. Finally, for the *TNFA* gene at positions −238G > A, −308G > A and −1031T > C, the most frequent alleles were G (94.32 %), G (86.17 %) and T (75.18 %).

**Table 2:** Levels of IgG antibodies against *P. vivax* blood stage proteins according to the studied genotypes

Gene	SNP	PvAMA-1 (n = 135)	*P* value	PvDBP (n = 135)	*P* value	PvMSP-1_19_ (n = 128)	*P* value
*INFG*	+*874T* > *A*		0.19		0.40		0.42
	*AA*	1.36 (0.63–2.78)		1.58 (0.78–7.29)		2.92 (1.00–7.63)	
	*AT*	1.14 (0.51–2.18)		1.41 (0.77–4.73)		2.05 (0.49–6.64)	
	*TT*	2.50 (1.18–3.00)		5.17 (0.86–18.5)		4.66 (1.50–8.11)	
*TNFA*	−*1031T* > *C*		0.74		0.42		0.71
	*TT*	1.41 (0.60–2.97)		1.34 (0.79–5.38)		3.74 (0.96–7.57)	
	*TC*	1.49 (0.58–2.50)		1.66 (0.79–6.87)		2.60 (0.51–6.93)	
	*CC*	1.45 (1.01–1.52)		8.57 (0.73–14.89)		2.12 (1.32–2.59)	
*TNFA*	−*308G* > *A*		0.41		0.58		0.50
	*GG*	1.67 (0.65–2.80)		1.66 (0.79–6.30)		2.92 (0.89–7.58)	
	*GA*	1.14 (0.58–2.23)		1.48 (0.73–3.42)		2.61 (1.18–6.54)	
	*AA*	0.62 (0.51–0.62)		1.39 (0.96–1.39)		1.30 (0.28–1.30)	
*TNFA*	−*238G* > *A*		0.76		0.97		0.61
	*GG*	1.45 (0.64–2.66)		1.59 (0.81–5.59)		2.61 (0.94–7.47)	
	*GA*	1.34 (0.53–2.91)		1.48 (0.76–7.16)		5.15 (1.69–8.16)	
	*AA*	2.32 (2.32–2.32)		1.28 (1.28–1.28)		3.04 (3.04–3.04)	
*IL10*	−*819C* > *T*		0.79		0.57		0.99
	*CC*	1.35 (0.63-2.53)		2.01 (0.79–5.91)		3.50 (0.55–7.48)	
	*CT*	1.45 (0.60-2.77)		1.29 (0.77–5.06)		2.61 (0.87–7.60)	
	*TT*	2.22 (0.83–2.84)		2.18 (1.35–6.31)		1.98 (1.22–7.13)	
*IL10*	−*592C* > *A*		0.86		0.86		0.77
	*CC*	1.41 (0.62–2.50)		1.59 (0.76–5.91)		4.92 (1.10–7.63)	
	*CA*	1.52 (0.62–2.78)		1.46 (0.81–5.46)		2.59 (0.94–7.26)	
	*AA*	1.22 (0.52–2.85)		1.84 (1.21–4.32)		2.50 (1.07–5.97)	

Values are presented as medians (IQ)

Antibodies levels (PvAMA-1, PvDBP or PvMSP-1_19_) were significantly higher in individuals with malaria than in uninfected (Additional file 1). No significant differences were found in the levels of IgG antibodies against the PvAMA-1, PvDBP or PvMSP-1_19_ proteins in relation to the studied polymorphisms (Table 2, *P* > 0.05, Kruskal–Wallis test). It was possible to genotype some samples of patients with malaria to the IFNG (+874A > T) gene rs2430561. This result did not change in the regression analyses after adjusting for covariates affecting antibody levels (current infection status, previous history of malaria and gender). The logistic regression analyses also revealed no significant differences between haplotypes in relation to antibody levels (Table 3).

**Table 3:** Frequency of haplotypes and their association with levels of IgG antibodies against the PvAMA-1, PvDBP and PvMSP-1_19_ proteins

Haplotypes	Frequency	PvAMA-1			PvDBP			PvMSP-1_19_		
		RI estimation	Difference (95 % CI)	*P* value	RI estimation	Difference (95 % CI)	*P* value	RI estimation	Difference (95 % CI)	*P* value
TNF_−1031/−308/−238_										
T/G/G	0.606	1.76	Reference		4.01	Reference		4.35	Reference	
C/G/G	0.201		−0.13 (−0.49 to 0.24)	0.50		1.86 (−0.15 to 3.86)	0.07		−0.62 (−1.75 to 0.52)	0.29
T/A/G	0.127		−0.31 (−0.74 to 0.12)	0.15		−0.89 (−3.20 to 1.41)	0.45		−0.58 (−1.88 to 0.73)	0.39
C/G/A	0.041		−0.07 (−0.78 to 0.63)	0.84		−1.51 −5.17 to 2.15)	0.42		0.19 (−1.99 to 2.36)	0.86
IL10_−819/−592_										
C/C	0.644	1.54	Reference		4.77	Reference		4.13	Reference	
T/A	0.311		0.05 (−0.25 to 0.35)	0.74		−0.09 (−1.83 to 1.66)	0.92		−0.48 (−1.42 to 0.46)	0.32
T/C	0.037		0.50 (−0.17 to 1.18)	0.14		−1.50 (−5.43 to 2.43)	0.45		1.34 (−0.75 to 3.44)	0.21

The effects of each haplotype were relative to the most frequent haplotype, which was used as the reference

Δ % indicates the relative change in antibody levels compared to reference haplotypes, with a confidence interval of 95 %

## Discussion

Cytokines such as TNF, IFN-γ and IL-10 participate in cellular and humoral immune modulation in malaria and affect pathogenesis, parasitaemia control and pathophysiology, which are dependent on the cellular and circulating levels of these cytokines [3, 21, 29, 30]. It is hypothesized that polymorphisms in the genes encoding these cytokines could be found in genetic markers and would affect the levels of IgG anti-merozoite antibodies in individuals with vivax malaria. Limited data are available in the literature, and studies examining associations between antibodies and SNPs in cytokine genes in general have been conducted only for *P. falciparum*. The aim of the present study was to evaluate the importance of polymorphisms in the genes *TNFA*, *IFNG* and *IL10* in the antibody response of *P. vivax* vaccine candidate proteins. However, analysis identified no significant association.

IFN-γ is a key pro-inflammatory cytokine for the induction of essential immune effector mechanisms in initial infection control in both the hepatic and erythrocytic phases of malaria [31, 32]. The production of this cytokine is related to low parasitaemia in the acute phase; however, a balance with anti-inflammatory cytokines, such as IL-10 and TGF-beta, is necessary to reduce severe forms of malaria [32]. The SNP at position +874 T > A affects an NFkB pathway that determines the production of inflammatory cytokines [33, 34]. An association has been observed between the T allele of this SNP and high IFN-γ production [34]. The results of individuals carrying the TT (Table 2) genotype had higher levels of anti-PvAMA-1, -PvMSP-1_19_ and -PvDBP IgG antibodies than those with the TA or AA genotypes, although the association was not significant. Thus, the TT genotype may not be related to increased production of IFN-γ, which would negatively modulate the Th2 immune response and antibody production in the analysed samples, given that these patients had higher antibody levels.

TNF participates in the total IgG response, which is mediated by follicular dendritic cells and dependent on soluble TNFR1 signalling [35], and high levels of this cytokine are related to malarial paroxysm [32] and severe malaria [6]. The A allele of *TNFA* (−308, −238) has been associated with elevated levels of antibodies in falciparum malaria [6, 35, 36]. At positions −863 and −857, the A and T alleles, respectively, have been associated with high levels of IgG3 and IgG4 antibodies in malaria [37]. In Tanzania, Carpenter et al. [11] reported a negative association between levels of anti-*P. falciparum* IgG antibodies and the A allele (−308) in malaria patients. In a study of SNPs in the *TNFA* gene in Burkina Faso, the A (−863), T (−857) and G (−1304) alleles in particular were associated with total IgG levels against *P. falciparum*; however, no association was found for positions −1031, −308 or −238 of this gene [6]. This prevalence of subjects individuals with the A allele (*TNF*308G > A) had the lowest levels of anti-PvAMA-1, -PvMSP-1_19_ and -PvDBP IgG antibodies, but the association was not significant.

Interleukin 4, IL-10 and IL-13 are anti-inflammatory cytokines involved in antibody production mechanisms [3]. IL-10 is a Th2-type immunoregulatory cytokine that negatively modulates the effects of pro-inflammatory cytokines produced by Th1 cells [29, 30, 38], participates in the induction of immunoglobulin synthesis and promotes isotype class switching from IgM to IgG1 and from IgG1 to IgG3 [6]. The AA genotype (−1082) has been associated with high levels of anti-MSP-2/31D7 and -AMA-1 IgG antibodies in mothers and newborns with falciparum malaria in Uganda [3]. In patients with falciparum malaria in Tanzania, the A allele (−592, −1082) was associated with low levels of IgE and IgG4 [11]. SNPs (−592 and −819) were associated with high levels of IgE and *P. falciparum* NANP (IgG) antigen antibodies in Sri Lanka [39]. Haplotypes IL10−1082/−819/−592 GCC, ACC and ATA were correlated with high, intermediate and low levels, respectively, of the IL-10 cytokine [29, 40]. It has been hypothesized that high cytokine concentrations are associated with the presence of the C allele at positions −819 and −592 based on the observation that IL-10 participates in the immunological activation of antibody production. However, this proposition was supported for the analyses carried out only for anti-PvMSP-1_19_ IgG for these genotypes. The opposite occurred in the presence of the TT (−819) and AA (−592) genotypes associated with low production of anti-MSP-1_19_; however, there was no statistical significance in either case. This finding may be due to low serum concentrations of the cytokine resulting from other factors or to the non-influence of these SNPS on IL-10 levels and antibodies in the patients evaluated in this study.

## Conclusions

This study revealed no association between genotypes and alleles with IgG class *P. vivax* anti-merozoite antibody levels. Studies of possible associations between SNPs in cytokine genes and the humoral immune response to malaria are still incipient, and the results are contradictory. However, this was the first Brazilian study to examine this set of SNPs (*IFNG*-*183G* > *T,* +*874A* > *T; TNFA* −*238G* > *A,* −*308G* > *A and* −*1031T* > *C; IL10*-*592C* > *A,* −*819C* > *T*) in control cases of vivax malaria. Immunogenetic profile studies are needed to better understand the immunomodulation of *P. vivax*; this research will be essential for the development of new malaria vaccines and treatments.

## Additional file

10.1186/s12936-016-1414-3 IgG antibody levels to PvAMA-1, PvDBP and PvMSP-1_19_.

## Abbreviations

BL: lymphocytes B
BLYS: B-lymphocyte stimulator
CD40: CD40 gene
CD86: CE86 gene
DARC: duffy antigen receptor for chemokines
ELISA: enzyme-linked immunosorbent assay
HLA-DR3: human leukocyte antigen-DR3
HLA-DR16: human leukocyte antigen-DR16
IgE: immunoglobulin E
IgG: immunoglobulin G
IgG1: immunoglobulin G, subclass 1
IgG2: immunoglobulin G, subclass 2
IgG4: immunoglobulin G, subclass 4
IL4: interleukin 4
IL-10: interleukin 10
*IL10*: interleukin 10 gene
IL-13: interleukin 13
*INFG*: interferon gamma gene
INFγ: cytokine interferon gamma
LTCD4: lymphocyte CD4 T
MSP-2/31D7: merozoite surface protein–2/31D
PvAMA-1: apical membrane antigen-1
PvDBP: duffy binding protein
PvMSP-1_19_: merozoite surface protein-1
RFLP: restriction fragment length polymorphism
RI: reactivity index
TNF: cytokine tumour necrosis factor
*TNFA*: tumour necrosis factor gene
VK247: variant of *Plasmodium vivax*
