# Supplementary material for: Cytokine gene polymorphisms are not associated with anti-PvDBP, anti-PvAMA-1 or anti-PvMSP-119 IgG antibody levels in a malaria-endemic area of the Brazilian Amazon
Source: Malar J. 2016 Jul 19;15:374. doi: 10.1186/s12936-016-1414-3 (PMC4952271; doi:10.1186/s12936-016-1414-3)
Supplement: Supplementary file 1 — 10.1186/s12936-016-1414-3 IgG antibody levels to PvAMA-1, PvDBP and PvMSP-119. [file 12936_2016_1414_MOESM1_ESM.docx]

Additional file 1. IgG antibody levels to PRIMA-1, PvDBP and PvMSP1-19.

| IgG antibody levels determined by reactivity index (IR) | | | |
| --- | --- | --- | --- |
| Antigens | Mild vivax malaria  (n = 90) | Uninfected  (n = 51) | *p* values |
|  |  |  |  |
| PvAMA-1 | 2.19 (0.93-3.04) | 0.69 (0.50-1.30) | < 0,01 |
| PvDBP | 2.75 (0.90-7.27) | 0.87 (0.64-1.82) | < 0,01 |
| PvMSP-1_19_ | 6.69 (2.86-8.11) | 0.87 (0.34-2.12) | < 0,01 |

IR: OD sample/OD cut-off value
